# Giant Leiomyoma of the Retzius Space: A Case Report

**DOI:** 10.1155/2013/371417

**Published:** 2013-03-25

**Authors:** Franco Pepe, Pietro Pepe, Filippo Rapisarda, Marta Fauzia, Maria Giunta

**Affiliations:** ^1^Obstetric and Gynecology Unit, Santo Bambino Hospital, Via Antico Corso 2, 95100 Catania, Italy; ^2^Urology Unit, Cannizzaro Hospital, 95100 Catania, Italy

## Abstract

Extrauterine leiomyoma is a very rare clinical condition; we report a case of leiomyoma of the Retzius space in a 49-year-old women who suffered for two years from bladder voiding symptoms characterized by dysuria, feeling of incomplete emptying, and pelvic pain. Clinical evaluation and abdominal and transvaginal ultrasound suggested the presence of a voluminous (about 10 cm in diameter) fibromyoma of the anterior uterus surface. The urodynamic evaluation demonstrated the presence of bladder outlet obstruction (voiding pressure greater than 20 cm H_2_O and maximum flow rate less than 12 mL/s) with a postvoiding urine residual equal to 80 mL; moreover, the presence of cystocele and urethral stricture was ruled out performing clinical evaluation, cystography, and cystourethroscopy. The patient underwent laparotomy to remove the uterine fibromyoma. Intraoperatively, a voluminous soft mass arising from the Retzius space was found; it was firmly adhered to the uterus with obliteration of vesicouterine pouch owing to severe adhesion to the anterior surface of uterus. The tumour was isolated, enucleated from the prevesical space, and removed; moreover, the patient became asymptomatic after surgery. In conclusion, leiomyoma of the Retzius space is a very rare benign tumour that should be considered in the presence of severe bladder voiding symptoms.

## 1. Introduction

Primitive tumours of the Retzius space are infrequently in women; although the neoplasms in most of the cases are characterized by benign histology, isolated cases of aggressive cancers of adjacent tissue (urachal remnant, vulva) can involve the Retzius space [1, 2]. 

The tumours may remain asymptomatic for a long time becoming very large when detected. Common symptoms are related to urinary voiding secondary to bladder compression; they include dysuria, frequency, or hesitance in urination, urinary retention. 

A rare case of a giant mass of the Retzius space diagnosed in a woman with urinary voiding symptoms is herein reported.

## 2. Case Report

 A 49-year old woman presented with a clinical history dated from two years of bladder voiding symptoms characterized by dysuria, feeling of incomplete emptying, and pelvic pain. On physical examination the vaginal examination, was painless and uterus was fixed with an irregular surface due to a voluminous anterior mass. The abdominal and transvaginal US showed normal ovaries and a mass of 10 cm in diameter localized in the anterior zone of the uterus; the ultrasound pattern suggested the presence of a uterine fibromyoma that compressed the bladder wall. The urodynamic evaluation demonstrated the presence of bladder outlet obstruction (voiding pressure greater than 20 cm H_2_O and maximum flow rate less than 12 mL/s) with a postvoiding urine residual equal to 80 mL; moreover, the presence of cystocele and urethral stricture was ruled out by performing clinical evaluation, cystography and cystourethroscopy.

**Figure 1:**
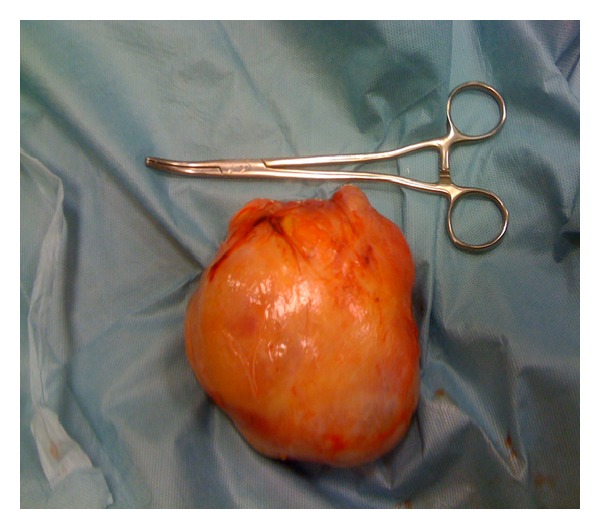
Giant leiomyoma (10 cm in diameter) of the Retzius space.

The patient underwent laparotomy to remove the uterine fibromyoma; a soft mass was found in the retropubic and prevesical space that was firmly adhered to the uterus with obliteration of vesicouterine pouch owing to severe adhesion to the anterior surface of uterus. The mass had a diameter of about 10 cm and rose from the space of Retzius; moreover, uterus, ovaries, and tubes were normal. The tumour was isolated, enucleated from the prevesical space, and removed (Figure 1). The postoperative course was uneventful and the patient was discharged from hospital four days from surgery. Definitive histological specimen showed the presence of a leiomyoma; moreover, the women became free of symptoms 1 month from surgery. 

## 3. Discussion

The majority of benign tumours of the Retzius space arise from connective tissue (granuloma, hemangiopericytoma, subpubic cartilaginous cyst, neurinoma, and lymphangioma) [3, 4] and are provided of a good prognosis. 

leiomyoma of the Retzius space constitutes a rare cause of urinary voiding difficulties due to obstruction of the urethra or/and bladder [5], and uptill today only five cases have been reported in the literature. Reisenauer et al. [6] reported a case of leiomyoma in a woman with voiding difficulties; Granados et al. [7] described a giant leiomyoma arising from the space of Retzius that compressed the bladder extending to the anterior abdominal wall; finally, Stutterecker et al. [8] report 2 women with a leiomyoma in the pubovesical space in whom the voiding symptoms reversed after surgery.

Preoperatively, clinical evaluation and US, as first choice, in most of the cases are not able to distinguish tumours of the Retzius space from the most common fibromyoma of the uterus; therefore, the indication to perform a more accurate radiological imaging (MRI or CT scan) for the suspicion of a pelvic mass often remains unheeded and only intraoperatively the right diagnosis is performed.

In conclusion, leiomyoma of the Retzius space is a very rare benign tumour that should be considered in the presence of severe bladder voiding symptoms.
